# Strengths and Weaknesses of the Current Strategies to Map and Characterize R-Loops

**DOI:** 10.3390/ncrna4020009

**Published:** 2018-03-27

**Authors:** Vincent Vanoosthuyse

**Affiliations:** UMR5239 CNRS, Université de Lyon, ENS-Lyon, 46 allée d’Italie, 69007 Lyon, France; vincent.vanoosthuyse@ens-lyon.fr

**Keywords:** R-loops, S9.6, RNase H1, DRIP, R-ChIP

## Abstract

R-loops are evolutionarily conserved three-stranded structures that result from the formation of stable DNA:RNA hybrids in the genome. R-loops have attracted increasing interest in recent years as potent regulators of gene expression and genome stability. In particular, their strong association with severe replication stress makes them potential oncogenic structures. Despite their importance, the rules that govern their formation and their dynamics are still controversial and an in-depth description of their direct impact on chromatin organization and DNA transactions is still lacking. To better understand the diversity of R-loop functions, reliable, accurate, and quantitative mapping techniques, as well as functional assays are required. Here, I review the different approaches that are currently used to do so and to highlight their individual strengths and weaknesses. In particular, I review the advantages and disadvantages of using the S9.6 antibody to map R-loops in vivo in an attempt to propose guidelines for best practices.

## 1. Introduction

R-loops form when a complementary RNA molecule invades the DNA double helix and hybridizes with one strand through Watson–Crick pairing, leaving the second DNA strand single-stranded. It is believed that most R-loops form in a co-transcriptional manner, when the nascent RNA invades its template in the wake of the RNA polymerase (reviewed in [1,2,3]). In eukaryotes, R-loops can form both at protein-coding and non-coding transcription units, whatever the RNA Polymerase involved and it is estimated that 5% of the human genome has the potential to form detectable R-loops [4]. R-loop biology is the focus of growing scrutiny, as increasing evidence suggest that the defective control of R-loop formation is associated with a number of human diseases [5]. Many questions remain however regarding the direct consequences of R-loop formation on the surrounding chromatin. 

Current data demonstrate that specific features facilitate R-loop formation. These include negative topological stress in the DNA, a strong bias for Guanine residues (G-skew) or the presence of homopolymeric Adenine tracts in the RNA sequence, and high rates of transcription (reviewed in [2]). Some of these features are inter-dependent, as high rates of transcription will lead to greater negative topological stress, and, incidentally, will also increase the likelihood of forming and hence detecting R-loops. Nevertheless, a consensus is emerging that the beginning of G-skewed genes represents strong R-loop forming regions [4,6,7,8], although it could be that those particular R-loops are thermodynamically more stable because of the G-skew, and hence easier to detect. Using some mapping methods at least, R-loops have also been shown to form in gene bodies and in terminator regions [4], although the presence of R-loops in gene terminators was not significantly detected in *Arabidopsis thaliana* [8]. Recent observations have suggested that promoter R-loops tend to form over DNA sequences where RNA Polymerase II is frequently in pause [6,9,10] and that have the potential to form G-quadruplex structures [6]. It is possible that R-loops, by displacing G-rich single-stranded DNA (ssDNA), facilitate the formation of intra-molecular G-quadruplexes, which in turn, could stabilize R-loops. Taken together, these observations clearly show that there are strong sequence determinants to R-loop formation. 

In eukaryotes, chromatin structure (i.e., the position and dynamic of nucleosomes) also modulates R-loop formation [11,12,13], presumably because the presence of nucleosomes counter-acts the propensity of the double-helix to open and be invaded by the nascent RNA. The folding of the nascent RNA and its coating by RNA-binding proteins such as the spliceosome is also believed to strongly antagonize R-loop formation, most likely by physically preventing the nascent RNA from threading back into its DNA template [14,15]. Consistent with this, the presence of introns was shown to limit R-loop formation [15] and conversely, R-loop formation was shown to increase significantly in many mutants of the RNA processing machinery [16,17]. Note however that, somewhat in apparent contradiction with this simple model, there is evidence that the homologous recombination (HR) machinery is essential for R-loop formation in many RNA processing mutants in budding yeast, suggesting that R-loop formation is an active process that requires the Rad51 recombinase [18]. The significance of this observation is not yet fully understood. More importantly, recent data suggest that DNA replication has a strong influence on R-loop formation [19,20]: head-on conflicts between the transcription and replication would facilitate R-loop formation, possibly because topological constraints associated with such conflicts induce more negative topological stress over R-loop forming genes, whilst co-directional conflicts would reduce R-loop formation, possibly because replisome-associated helicases could disassemble R-loops. In addition, recent data suggest that in human cells at least, R-loop formation in mitosis is largely restricted to centromeres [21]. Taken together, these observations strongly suggest that the cell cycle stage greatly influences the abundance and localization of R-loops. To sum up, although our understanding of the features that modulate R-loop formation has increased considerably in recent years, in-depth and consensual mechanistic details of R-loop formation at the molecular level are still missing.

R-loops have been previously proposed to act as double-edged swords in the genome [1], because they can be both physiological intermediates in some key processes and pathological structures. For example, R-loops have been shown to participate in class-switch recombination at immunoglobulin genes [22,23], in the initiation of mitochondrial replication [24], in the hypo-methylation of promoters [25,26] or in transcription termination [27,28,29]. Recently, R-loops have also been proposed to form on either side of double-strand breaks [30]. Conversely, R-loops are mutagenic [31], they stimulate transcription-associated recombination (TAR) [32], and they are associated with DNA damage in a replication-dependent manner [33]. In particular, R-loops are hugely detrimental in head-on collisions between transcription and replication, where they lead to severe genome instability [19,20]. What features determine when R-loops become pathological structures remains poorly understood, but there is general agreement that R-loop stability and the chromatin context in which they form are likely to be key contributing factors. It is also possible that there are in fact different types of R-loops in the cell, depending, for example, on their size or their sequence, and that those different R-loops have different levels of toxicity depending on their architecture or the set of proteins that recognize them. Consistent with the idea that there could be different types of R-loops, R-loops that form in the absence of the THO complex in *Saccharomyces cerevisiae* display much higher levels of TAR than those that form when both ribonuclease (RNase) H1 and H2 are missing [32].

To address further the possible functions of R-loops, reproducible, accurate, and quantitative R-loop mapping techniques are needed. The most widely used of these mapping techniques is DNA:RNA immunoprecipitation (DRIP) [25] that relies on the use of the S9.6 antibody, which recognizes DNA:RNA hybrids with great affinity [34]. Recently, several studies have questioned the robustness of this approach [6,7,35] and an alternative strategy to map R-loops has been developed [6]. In addition, the understanding of R-loop functions necessitates robust and specific approaches to modulate R-loop formation. For this, the available toolkit remains very limited and most studies rely on the nuclear over-expression of RNase H, an enzyme that destroys R-loops without specificity, whether they are physiological or pathological.

Here, I aim to conduct an impartial assessment of the strengths and weaknesses of the different methods to map and evaluate the functions of R-loops. Based on these observations, I propose guidelines for best practices when working with R-loops.

## 2. Non-Denaturing Bisulfite Footprinting to Map R-Loops

Exposure of single-stranded DNA (ssDNA) to sodium bisulfite converts unmethylated cytosine residues into uracil and this property has been used to map the ssDNA that is associated with R-loops [7,22]. In a non-denatured R-loop, only the unmethylated cytosine residues present in the non-template strand should be converted, as those in the template strand are protected from conversion by the hybridized RNA. The presence of an R-loop is confirmed by the fact that cytosine conversion is abolished after exogenous RNase H treatment. The great advantage of this approach is that it allows for the footprinting of R-loops at near nucleotide resolution and at the single molecule resolution. For a given locus, this technique gives access to the distribution of R-loop sizes and positions. Its main limitation, however, at least in its current state, is that it is very low-throughput and that its resolution is strongly constrained by the number and position of unmethylated cytosine residues in the non-template strand. For instance, this approach would be inappropriate to map R-loops forming within polyA tracts [36]. Nevertheless, it is tempting to speculate that the development of novel long-read and single-molecule sequencing technologies, such as PacBio single-molecule real time (SMRT) sequencing could soon make the genome-wide bisulfite mapping of R-loops a reality. Alternatively, to map R-loop associated ssDNA, the use of the ssDNA-binding protein replication protein A (RPA) could be envisaged. However, to be precise and specific for R-loops, stranded and quantitative RPA chromatin immunoprecipitation (RPA-ChIP) would have to be set up to precisely identify RNase H-sensitive RPA-ChIP signals.

## 3. The S9.6-Based Methods to Map and Quantify R-Loops

To date, most of the methods to quantify and map R-loops both in vitro and in vivo rely on the use of the S9.6 monoclonal antibody [34]. S9.6 recognizes predominantly DNA:RNA hybrids, although several studies have shown that it has some weaker, but nevertheless significant affinity for RNA:RNA hybrids as well [37,38,39]. In addition, it was proposed that the affinity of S9.6 for DNA:RNA hybrids is influenced by their nucleotide composition and that this might introduce bias in S9.6-based maps [35]. Nevertheless, S9.6 is classically used for dot blot analysis, immunofluorescence studies and immunoprecipitation approaches.

It is important to note that, besides the technical challenges and the potential limitations that are associated with using S9.6 (see below), S9.6 should recognize any DNA:RNA hybrids greater than six nucleotides in length [37] in the genome, whether they correspond to R-loops or to DNA:RNA hybrids produced during normal DNA transactions, such as DNA damage repair or Okazaki fragments synthesis. Theoretically at least, both genuine R-loops and other DNA:RNA hybrids should yield RNase H-sensitive S9.6 signals. In practice, this could be a problem for example when mapping DNA:RNA hybrids at stalled replication forks, where it is conceivable that the RNA primers required to synthesize Okazaki fragments could be stabilized and might give significant S9.6 signals. Therefore, unless one works with synchronized populations of cells, it is possible that DNA:RNA hybrids other than R-loops will contribute to the signals obtained using S9.6-based methods. At this point, one should stress that this is still only a theoretical limitation of using S9.6 to characterize R-loops, but one that seems important to keep in mind and that should be carefully investigated in the future.

### 3.1. Quantifying R-Loop Formation Using Dot Blots

When properly controlled using RNase H treatment and accurate DNA quantification, dot blot analysis is a reliable and straightforward way of quantifying DNA:RNA hybrids [33,40,41]. Note however, that, unless nucleic acids have been extracted from purified nuclei, the material that is probed tends to be mostly a mixture of genomic, mitochondrial, and in some cell-types, cytosolic DNA. As a result, dot blot analysis is probably only sensitive enough to detect major changes in R-loop accumulation but not to detect small, localized changes in R-loop formation in genomic DNA.

### 3.2. Visualizing R-Loop Formation Using Immunofluorescence Approaches

S9.6 has been used extensively in immunofluorescence studies, both on whole cells and on chromosome spreads. On whole cells especially, the available results are somewhat confusing because the pattern obtained with S9.6 varies significantly from one study to another (see below). There might be cell-type specificities to explain this varying pattern, but it is more likely, as shown by Skourti-Stathaki et al., that the conditions used to fix the cells have a strong influence on the S9.6 pattern [28]. Note however, that to date, no objective criteria have been established to help decide which fixating condition best preserves genuine R-loops.

Most studies detect strong cytoplasmic and nucleolar signals with the S9.6 antibody, whilst the 4’,6-diamino-2-phenylindole (DAPI)-stained nuclear signal is surprisingly weak in comparison. Although the nucleolar signal could be explained at least in part by the well-described and evolutionarily conserved formation of R-loops at the ribosomal DNA (rDNA) [42,43,44], a very strong cytoplasmic signal is unexpected and somewhat confusing. In some studies, this cytoplasmic signal is restricted to a region around the nucleus that is known to be rich in mitochondria. In the majority of studies, however, the cytoplasmic signal is much more widespread.

The cytoplasmic signal is often attributed to R-loops produced in the mitochondria [25], but others have disputed that fact by showing that there is no significant co-localization between the S9.6 signal and mitochondria [45]. Koo et al. proposed instead that the cytoplasmic signal that is detected by S9.6 comes for a large part from cytosolic DNA:RNA hybrids whose RNA moiety are produced by RNA polymerase III (RNAPIII) [45]. Note however that the origin of the DNA in these putative cytoplasmic RNAPIII-dependent DNA:RNA hybrids remains mysterious. Intriguingly, a strong cytoplasmic signal was also obtained when cells were stained with the J2 antibody that recognizes RNA:RNA hybrids [28]. As S9.6 has a significant affinity for RNA:RNA hybrids [37,38,39], it is possible that at least part of the cytoplasmic signal detected with S9.6 can be attributed to highly structured RNAs or even RNA:RNA hybrids. The same can be said for the nucleolar signal because the nucleolus also contains a lot of hairpin-forming RNAs. These observations suggest that the S9.6 pattern after immunofluorescence on whole cells might result from both R-loop and non R-loop structures. 

Because the cytoplasmic signal obtained with S9.6 is not fully understood, it is often ignored, even if it is much stronger than the nuclear signal. Surprisingly, however, there are many mutant conditions where the S9.6 signal increases both in the cytoplasm and in the nucleus, even when proteins that are thought to be exclusively nuclear are mutated: for example, the deficiencies of BRCA2 [10,46], Senataxin [47], U2 snRNP [48], SRPK2, and DDX23 [49] all lead to a significant increase in cytoplasmic S9.6 staining. This counter-intuitive observation remains unexplained. One possible explanation is that S9.6 detects a stress-associated structure that is found in the cytoplasm and perhaps also in the nucleus and the nucleolus. If this is true, quantifying the nuclear S9.6 signal by immunofluorescence as a measure of R-loop formation is probably inaccurate. Finally, these observations suggest that S9.6 probably recognizes other structures than DNA:RNA hybrids in situ and that its specificity for DNA:RNA hybrids is very sensitive to the way the DNA/chromatin is fixed or extracted.

What steps could be taken to improve this otherwise convenient approach to monitor R-loop formation in situ? First, it seems imperative that the wider community should quickly agree on a fixation protocol to best preserve R-loops and their sensitivity to RNase H. Then, the quantification of the cytoplasmic, nucleolar and nuclear S9.6 signals and their respective sensitivity to exogenous RNase H treatment should be systematically presented in a statistically significant number of cells. In addition, a small improvement to a previously developed non S9.6-based strategy could be implemented in parallel for corroboration: the Aguilera lab used the 52-residue DNA:RNA hybrid-binding domain of RNase H1 (HB-GFP) to quantify R-loops in vivo after cell permeabilization [46]. An additional control for this approach could be to express a mutated version of HB-GFP that cannot recognize DNA:RNA hybrids anymore (see below). Note, that as the over-expression of HB-GFP might interfere with the binding of the endogenous RNase H1 to R-loops, it is possible that HB-GFP over-expression might artificially stabilize R-loops. When implementing this strategy, it is therefore essential to show that the expression levels of HB-GFP are comparable in the different backgrounds/conditions where R-loops are to be quantified.

### 3.3. DNA:RNA Immunoprecipitation (DRIP) and DRIP-Like Methods

**Figure 1:**
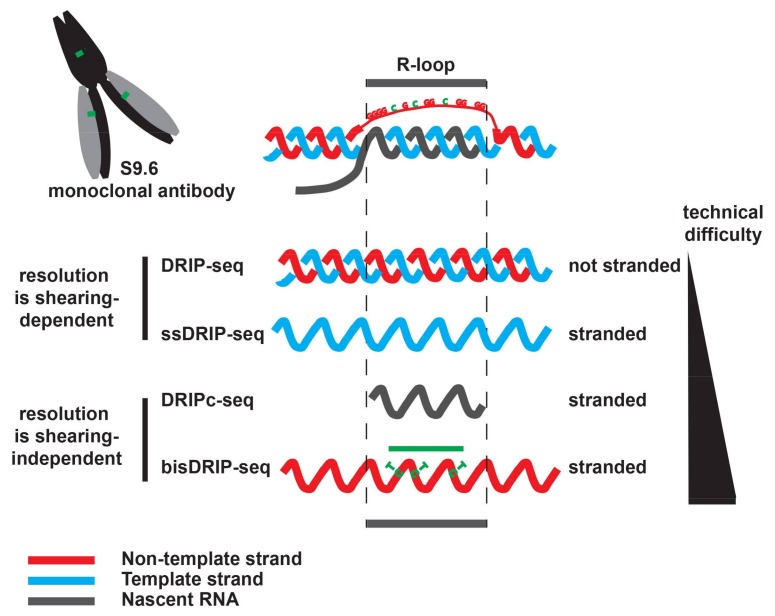
Genome-wide mapping of DNA:RNA hybrids using DNA:RNA immunoprecipitation (DRIP)-like approaches.

The use of the S9.6 antibody to map DNA:RNA hybrids at specific loci was first developed by the Tollervey and the Proudfoot labs in yeast [44] and in human cells [27], respectively. A similar strategy was then implemented by the Chédin lab to obtain genome-wide maps of RNase H-sensitive R-loops in human cells [25]. The Chédin lab coined the now widely-used name DNA:RNA immunoprecipitation (DRIP) to describe this approach. The principle of DRIP is very simple: nucleic acids are extracted and sheared, and the DNA:RNA hybrids are immuno-precipitated using the S9.6 antibody. Pre-treatment of half the sample with exogenous RNase H validates the specificity of the immunoprecipitation, although this crucial control is not always shown (see below). When studying a small set of loci, the enrichment of DNA:RNA hybrids is classically estimated using quantitative polymerase chain reaction (DRIP-qPCR). To better demonstrate the presence of R-loops, a few studies have used reverse transcription qPCR (RT-qPCR) on DRIP material (DRIP-RT-qPCR) to definitely demonstrate that the immuno-precipitated material did indeed contain RNase H-sensitive RNA molecules [4,8]. At the genome-wide scale, various sequencing techniques have been implemented (Figure 1): DRIP-seq sequences the DNA present in the immuno-precipitated material [25]; for increased resolution, DRIPc-seq sequences that the RNA molecules present in the immuno-precipitated fraction [4,38]; ssDRIP-seq sequences the template strand hybridized to the R-loop RNA [8]; bisDRIP-seq combines S9.6 immunoprecipitation with bisulfite footprinting to map the R-loop-associated ssDNA [7]. DRIPc-seq and bisDRIP-seq map R-loops in a strand-specific manner at near nucleotide resolution. Although DRIP has considerably improved our understanding of R-loops in recent years, it suffers from an apparent lack of robustness between studies, which begs some questions.

After DNA extraction and shearing, the S9.6 monoclonal antibody is used to precipitate DNA:RNA hybrids. After purification, the precipitated DNA:RNA hybrids are sequenced. DRIP-seq ([25], not stranded) and ssDRIP-seq ([8], stranded) sequences the associated DNA. With these strategies, DNA:RNA hybrids are mapped with a resolution that is dependent on the size of the starting DNA fragments, which could be bigger than the R-loop. DRIPc-seq sequences the RNA moiety in the DNA:RNA hybrid in a strand-specific manner ([4,38]). Paired-end sequencing of the RNA ensures that the exact borders of the sequenced RNA are known. As a result, DRIPc-seq maps DNA:RNA hybrids in a strand-specific manner at near nucleotide resolution. Because it requires working with RNA, its implementation and the building of the sequencing libraries is technically more challenging than DRIP-seq. bisDRIP-seq [7] maps R-loops by combining the precipitation of the DNA:RNA hybrids with S9.6 with the bisulfite footprinting of the R-loop associated ssDNA. It is stranded (see text), but its resolution depends on the number of unmethylated cytosine residues that are present in the non-template strand and as a result it might under-estimate the size of R-loops (green line). However, with bisDRIP-seq, the R-loop associated ssDNA is modified before DNA extraction, which could limit the loss of unstable R-loops that might happen during DNA extraction. Of those four approaches, bisDRIP-seq is probably the hardest to implement and to analyze.

#### 3.3.1. Apparent Lack of Robustness of the DRIP Method

DRIP in different studies sometimes gives inconsistent results. For example, in *S. cerevisiae*, R-loops were shown by some [50] but not others [51] to accumulate at PMA1 when the DNA&RNA helicase Sen1 is deficient. The pattern of R-loop formation at the commonly-used gene model Actß varies also unexpectedly between studies: R-loops would be as likely to form within the first intron and the terminator of the gene for some [28,52], much more likely to form within the terminator for others [26], and finally far less likely for a fourth study [10]. Similarly, R-loops were shown by some to accumulate at terminator but not promoter regions when BRCA1 is deficient [10,52], whilst others detected R-loop accumulation particularly at promoter regions in those conditions [9]. In addition, DRIP enrichment for a given locus sometimes varies significantly from one study to the next or even within one study from one figure to another. At the genome-wide scale, there is also apparent variability in the number of R-loop forming regions that were identified using DRIP: for example, after DRIPc-seq, one study reported 8112 promoters forming R-loops forming regions in human embryonic carcinoma Ntera2 cells [25], whilst another DRIP study only identified 3257 R-loop forming promoters in human primary fibroblasts [26]. It is conceivable that at least some of these apparent discrepancies could be explained by the fact that cell types and hence transcriptional patterns often differ between studies, although there is evidence that patterns of R-loop formation are conserved between cell-types [4]. Moreover, different sequencing depths or peak calling algorithms could conceivably account for the inconsistencies in genome-wide DRIP studies. However, until it is firmly established that such parameters do indeed explain those differences, one is left feeling that, despite its very simple principle, DRIP is either not that straightforward to implement or not robust enough for its purpose. What could explain this apparent lack of robustness?

Many different DRIP protocols have been set up. Some of those protocols contain steps that are considered as very detrimental by others: for example some protocols include fixation and sonication steps [40], whilst both of these steps are to be absolutely avoided according to other protocols [36]. Although the aim of this review is not to discuss the different DRIP protocols, but rather to highlight the intrinsic strengths and weaknesses of the DRIP approach as compared to other R-loop mapping methods, it is likely that the different protocols and in particular the way the DNA is sheared and extracted could explain part of the variability that is observed. As explained below, other parameters could also contribute to this variability.

#### 3.3.2. Factors Contributing to the Lack of Robustness of DRIP

As discussed above, it is highly likely that the way the DNA is extracted has a very significant impact on the DRIP signal. In addition, the percentage of cells in S-phase in the starting material and the specificity of the antibody are also key factors to take into account to explain those discrepancies.

*The cell-cycle profile.* The recent observation that DNA replication is a modulator of R-loop accumulation [19,20] demonstrates that the cell-cycle profile of the starting material, and in particular, the proportion of cells in S-phase and in mitosis, will greatly influence the DRIP results. One should therefore either synchronize the samples of interest or endeavor to compare cultures with similar proportions of cells in S-phase and mitosis.

**Figure 2:**
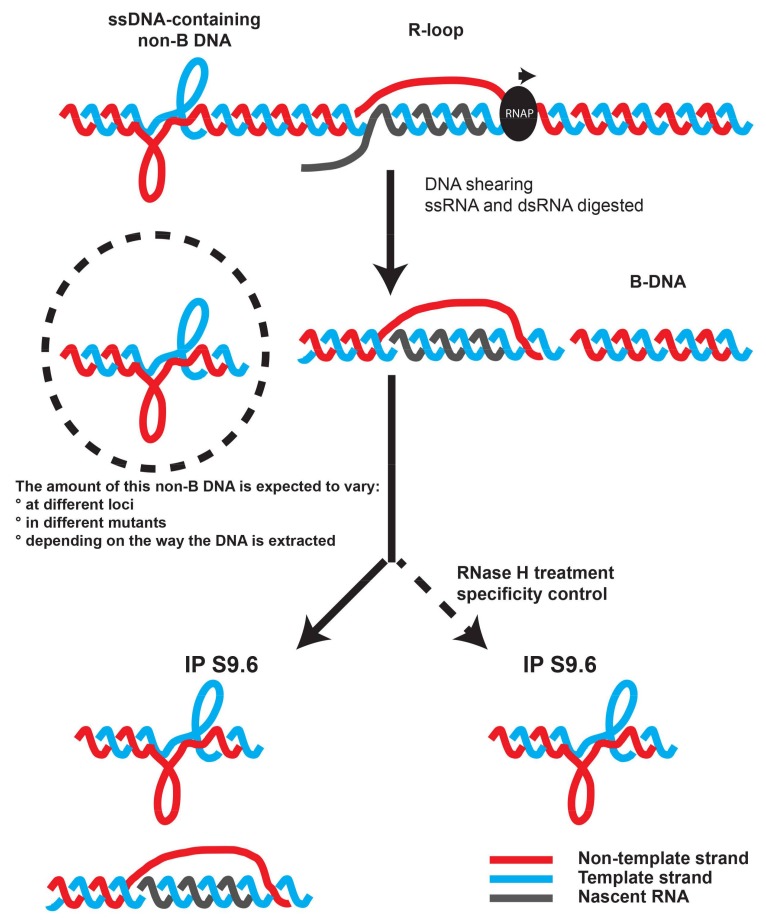
The S9.6 antibody is likely to recognize also ssDNA-containing non-B DNA forms that do not correspond to R-loops [7].

*The specificity of the antibody.* As mentioned above, although the greater affinity of the S9.6 antibody for DNA:RNA hybrids is very clear on in vitro substrates, several observations indicate that it can also recognize other structures when used on more complex material. For example, we recently showed that the known affinity of S9.6 for RNA:RNA hybrids, albeit weaker than its affinity of DNA:RNA hybrids, remained a confounding parameter for the accurate mapping of R-loops in fission yeast using DRIPc-seq [38]. In addition, the use of bisDRIP-seq has shown that S9.6 could efficiently immuno-precipitate single-stranded promoter regions that did not contain R-loops [7]. These observations strongly suggest that S9.6 is able to recognize a variety of nucleic acids that do not adopt the conformation of the ordinary B form of DNA. It is therefore conceivable that the way the genomic DNA is extracted will greatly influence the extent to which these other non-B forms of DNA are preserved, and hence the level of RNase H-resistant signal in DRIP experiments (Figure 2). This could at least partly explain the discrepancies between studies. DRIP enrichments might result from a combination of features recognized by S9.6 and might not necessarily only reflect the presence of R-loops. For example, the strong DRIP signal at the termination site (TES) of the *TEFM* gene in human fibroblasts is fully resistant to RNase H treatment [10]. This is why the RNase H-treated sample is such an important control, which cannot be replaced by complicated normalization methods as it is sometimes done. These observations strengthen the argument that the best way to demonstrate the presence of R-loops at a particular locus is to show the presence of strand-specific and RNase H-sensitive RNA using RNA-based methods, such as DRIP-RTqPCR or DRIPc-seq [4,8,38].

Scheme illustrating the fact that DRIP signals could result from R-loop and non R-loop structures and the importance of the RNase H treatment to accurately estimate DNA:RNA hybrid formation at a locus of interest.

## 4. RNase H1-Based Methods to Map R-Loops

RNase H1 is a highly conserved enzyme that recognizes DNA:RNA hybrids and cleaves their RNA moiety. In human cells, the low abundant RNase H1 is particularly enriched in mitochondria and in the nucleolus in a transcription-dependent manner [42]. Its role in the nucleus is not yet very well characterized however. As it is an endogenous activity that evolved to recognize and to disassemble DNA:RNA hybrids, RNase H1 has been used as a tool to map R-loops. In particular, several studies have used a mutant of RNase H1 that could recognize but not process DNA:RNA hybrids [6,25,43,53]. The earliest strategy was to express this catalytically inactive human RNase H1 (hRNase H1-D145N) in bacteria and to couple it to amylose beads. R-loops were then purified by affinity from sheared genomic DNA. This strategy, called DNA:RNA in vitro enrichment (DRIVE-seq), identified fewer R-loops than DRIP-seq [25,53]. To improve the sensitivity of this strategy, we made the same mutation in the endogenous RNase H1 enzyme in fission yeast (Rnh1-D129N), and we used chromatin immunoprecipitation (ChIP) of Rnh1-D129N to demonstrate the formation of unstable R-loops at tRNA genes [43]. The enrichment of Rnh1D129N at tRNA genes was fully sensitive to the strong in vivo expression of catalytically-active RNase H1 from *Escherichia coli* (RnhA), validating the use of Rnh1D129N as an R-loop reporter at these sites [43]. A similar approach was recently implemented in human cells using the nuclear over-expression of the catalytically inactive RNase H1-D210N and was renamed R-ChIP [6]. Importantly, the sequencing of R-ChIP reactions in this latest study was strand-specific and was controlled using a mutant of RNase H1 that cannot recognize DNA:RNA hybrids [6].

As with DRIVE-seq, R-ChIP identified fewer R-loop forming regions than the S9.6-based DRIP-seq or DRIPc-seq approaches [6]. Importantly, R-ChIP identified R-loops mostly at promoters, but not at terminator regions [6]. In addition, R-ChIP identified smaller R-loop forming regions than DRIPc-seq [6]. The size of R-loops identified by R-ChIP was very similar to the archetypical size of R-loops mapped in vitro at nucleotide resolution using non-denaturing bisulfite footprinting. Moreover, the R-loop forming regions identified by R-ChIP contained sequence motifs, such as clusters of Gs in the non-template strand that were previously identified in vitro as potential triggers for R-loop formation [54]. Finally, in both fission yeast and human cells, R-ChIP identified tRNA genes as hotspots of R-loop formation in otherwise wild-type cells [6,43], whilst tRNAs were identified as R-loop forming regions by DRIP in *A. thaliana* [8], but not in human cells [4,25]. Taken together, these observations led Chen et al. [6] to conclude that R-ChIP is better at identifying genuine R-loops than S9.6-based methods.

There are however significant down sides to R-ChIP. Although it is easy to implement in yeast [43], it is harder to implement in vertebrate cells, because it requires the stable expression of a mutant enzyme. As shown recently [21], the over-expression of a catalytically-inactive RNase H1 enzyme presents the risk of interfering with the dynamics of R-loops in vivo, when there is good evidence that R-loop dynamics is likely to be critical for gene expression and genome stability. To implement R-ChIP in mammalian cells, it is therefore important that the catalytically-inactive RNase H1 is expressed at the right level: too much expression and there is a risk of dominant-negative effects; too little expression and there is a risk that the endogenous, catalytically-active enzyme could interfere with the binding of the catalytically-inactive mutant and the efficiency of R-ChIP. More importantly even, there is a significant risk that R-ChIP is only going to map the R-loops that are recognized by RNase H1. As an increasing number of proteins have been postulated to recognize and disassemble R-loops in vivo (see for example Senataxin [55], FANCM [56], BLM [57], DDX19 [58], MTR4 [31] among others), it is conceivable that there might be different types of R-loops that could be recognized by different types of proteins. This might be why R-ChIP did not identify terminator regions as R-loop hotspots in human cells [6], where R-loops might be recognized and disassembled by DNA&RNA helicases, such as Senataxin and not by RNase H1 [27]. This could also explain why R-ChIP and DRIVE-seq identified fewer R-loop forming regions than DRIP-seq or DRIPc-seq.

We argued previously that R-ChIP and DRIP are complementary approaches because their use in parallel could give information about the stability of R-loops at specific loci [43]. For example in fission yeast, RNase H1 is most abundant at RNAPIII-transcribed genes, suggesting that R-loops are constantly formed and detected there [43]. The R-ChIP signal is therefore very strong at RNAPIII-transcribed genes and this was also true in human cells [6]. On the contrary, DRIP-qPCR and DRIPc-seq only gave significant signals at RNAPIII-transcribed genes in fission yeast in the absence of RNase H1 and RNase H2 [38,43], thus confirming that RNAPIII-transcribed genes produce R-loops that are efficiently degraded by RNase H enzymes. To summarize these observations, it is conceivable that DRIP is better suited at detecting long-lived R-loops, whilst R-ChIP could be better at detecting highly dynamic R-loops that are processed by RNase H1.

## 5. RNase H Over-Expression as a Tool to Probe R-Loop Functions

As discussed above, the direct consequences of R-loop formation on the surrounding chromatin are still largely unclear. An in-depth understanding of R-loop contribution to gene expression and genome stability necessitates functional assays where R-loop formation could be specifically modulated. Importantly, to secure an unequivocal interpretation of the data, R-loop formation should be modulated without interfering with transcript synthesis or integrity.

The most common functional assay to probe R-loop functions relies on the long-term modulation of R-loop levels by affecting RNase H activity in vivo: RNase H-sensitive R-loops are classically stabilized by deleting or down-regulating RNase H enzymes and in most model systems, RNase H-sensitive R-loops can be de-stabilized by artificially increasing the concentration of RNase H enzymes in the nucleus. When increasing (or decreasing) RNase H activity in the nucleus alters a phenotype of interest, it is concluded that R-loops contribute to this phenotype. Note however that this approach does not demonstrate that the contribution of R-loops to the phenotype of interest is direct or indirect.

The most widely used strategy is to over-express RNase H. Although being widely used, this strategy presents significant disadvantages. Its biggest limitation is that the amount of R-loops is reduced genome-wide and without specificity, meaning that both the physiological and the pathological RNase H-sensitive R-loops are affected. In addition, although this has never been shown, there is at least a theoretical risk that a strong concentration of RNase H in the nucleus might also interfere with the steady-state of other DNA:RNA hybrids, such as the primers of Okazaki fragments or the DNA:RNA hybrids that were recently detected at double-strand breaks during repair [30,59]. This could be why RNase H1 over-expression in human cells was associated with persistent DNA damage [42]. Finally, as discussed above, the possibility that there are RNase H-resistant R-loops in vivo that would resist such treatment has not yet been excluded.

Importantly, the fact that RNase H over-expression has an indiscriminate effect on DNA:RNA hybrids opens the possibility that it will have a significant impact on the transcriptome and the proteome, and that those changes could indirectly contribute to alter the phenotype of interest. If true, this would seriously complicate the interpretation of such experiments. For example, it was shown that the over-expression of RNase H1 in human cells significantly affects the protein levels of Top1 and other DNA repair proteins [42]. In addition, we recently demonstrated that the strong expression of *E. coli* RnhA in fission yeast had a significant impact on the transcriptome and affected the steady-state levels of many RNAs that did not form R-loops, according to our DRIPc-seq maps [38]. These observations confirmed that long-term manipulation of RNase H activity imparts significant and indirect changes to the transcriptome and proteome. In addition, the transcriptome and proteome alterations that are associated with RNase H over-expression are likely to differ in different genetic backgrounds. In particular, the modifications to the transcriptome imparted by RNase H over-expression could combine with the existing alterations to the transcriptome in some mutant backgrounds. Consistent with this, our unpublished results indicate that the strong expression of RnhA in fission yeast differently alters the transcriptome in different mutant backgrounds. Therefore, the fact that a phenotype of interest in a mutant background is sensitive to RNase H over-expression does not necessarily mean that R-loops contribute to this phenotype directly. Nevertheless, if this strategy were to be implemented to probe R-loop function because of its relative ease, it should probably be backed up by the demonstration that the over-expression of another R-loop removing enzyme also impacts the phenotype of interest in a similar way. Alternatively, ectopic expression of AID, a cytidine deaminase that targets cytosine residues in ssDNA, was shown previously to enhance R-loop dependent recombination and mutagenesis in yeast [13,60], and could be used to probe R-loop functions at the genome-wide level. Note however that AID could also target ssDNA present in other forms of non-B DNA [61], which could complicate the interpretation of such experiments.

However, if the consequences of R-loop formation on gene expression and genome stability are highly dependent on the chromatin context in which they form, locus-specific ways of manipulating R-loops must be developed. A corollary to what was discussed above is that locus-specific assays should be preferred to genome-wide approaches anyway because they are probably at lower risk of imparting indirect effects. To modulate R-loop formation locally, promoter-inactivating mutations [62] or ribozyme-induced transcript cleavage [32] have been used previously. The down side of these approaches is that they affect R-loop formation by interfering with the synthesis or the integrity of the transcript itself. Unless one provides strong arguments establishing that the sole purpose of transcription at the locus of interest is to form R-loops, the interpretation of such experiments can be complicated because they remove both the R-loop and the transcript. Ideally, one should aim to modulate the R-loop formation without affecting the synthesis of the transcript. To increase the concentration of RNase H (or other R-loop removing enzymes) locally using chromosome-targeting approaches could be an alternative strategy. A down side to this approach is that it is possible that the chromosome-targeting mechanism would interfere with the activity, the dynamics or efficiency of RNase H, which would result in only an incomplete reduction of R-loop levels. Another possibility could be to use genome editing to alter the sequence determinants leading to R-loop formation in a sequence of interest, without interfering with the synthesis of the transcript. Although this should be in theory the best approach, it also presents down sides: it is cumbersome to implement because the sequence determinants would have to be identified and validated in vitro before being mutated in vivo; in addition, it would be difficult to alter such sequence determinants in protein-coding sequences without altering the protein sequence itself, because some amino-acids, such as Glycine, are exclusively encoded by G-rich codons. Depending on the phenotype that is investigated, this could complicate the interpretation of the results. This approach might therefore be better implemented to understand the role of R-loop formation within non-coding transcription units.

To conclude, to go further forward in the in-depth characterization of R-loop contributions to gene expression and genome stability, there are some serious technical hurdles: RNase H over-expression, which is the strategy that is easiest to implement, is subject to potential indirect effects, whilst locus-specific strategies that would be better adapted at evaluating the direct consequences of R-loop formation on the surrounding chromatin are both risky and difficult to implement. Consensual proof-of-concept experiments are required to move the field forward.

## 6. Conclusions

This short survey of the strengths and weaknesses of the different methods used to characterize R-loop distribution and functions highlighted several important conclusions:

1. Current immunofluorescence approaches are unlikely to be a reliable way of quantifying R-loops.
2. The affinity of the S9.6 antibody for RNA:RNA hybrids and other non-B forms of DNA is potentially a confounding parameter for the quantification and the mapping of genuine R-loops using DRIP-like approaches.
3. The possible influence of the cell-cycle on the formation and/or stability of R-loops and the fact that the S9.6 antibody could theoretically recognize DNA:RNA hybrids associated with Okazaki fragments suggest that the cell-cycle profile of the starting material is a parameter to consider when performing DRIP.
4. More evidence is needed to demonstrate that R-ChIP is able to map all types of R-loops rather than a subset of abundant, G-skewed, promoter-associated R-loops.
5. Possible indirect perturbations to the transcriptome and proteome associated with prolonged manipulation of RNase H levels must be taken into consideration when using this strategy to characterize R-loop functions.

Several recommendations can be drawn from this. Because DNA replication has recently emerged as a significant modulator of DNA:RNA hybrids formation, it is important to know the proportion of cells undergoing DNA replication when quantifying R-loop formation in different mutant backgrounds (if one mutant spends more time in S-phase than the other, the abundance of DNA:RNA hybrids might be artificially skewed). Otherwise, and unless one is specifically interested in the role of R-loops during DNA replication, R-loops might be best quantified and mapped on populations of cells synchronized in G1 or G2. In any case, the genomic DNA should be pre-treated with RNases to remove both RNA:RNA hybrids and remnants of chromatin-associated RNAs [38]. Importantly, the wider community should quickly agree on a set of guidelines on how best to extract DNA to preserve R-loops. To improve resolution and demonstrate without doubt that the mapped R-loops do indeed contain hybridized RNA molecules, RNA-based methods such as DRIP-RTqPCR and DRIPc-seq are probably better. As an improvement to the current DRIPc-seq data and to reduce potential priming bias during retro-transcription of the hybridized RNA, adapter-based primers rather than random hexamers could be used, as is commonly done to sequence small RNAs. In addition, because R-loop formation is mainly a co-transcriptional event, methods should be developed to normalize DRIP or R-ChIP signals to nascent RNA transcription. When possible, R-ChIP and DRIP-like approaches could be used in parallel for corroboration as was done previously [43]. Finally, new locus-specific approaches should be developed to evaluate the direct consequences of R-loop formation on the surrounding chromatin.
